# Data on *HLA* class I/II profile in Brazilian pemphigus patients

**DOI:** 10.1016/j.dib.2016.05.077

**Published:** 2016-06-03

**Authors:** Maria José Franco Brochado, Daniela Francisca Nascimento, Neifi Hassan Saloum Deghaide, Eduardo Antonio Donadi, Ana Maria Roselino

**Affiliations:** a Division of Dermatology, Department of Clinical Medicine, Ribeirão Preto Medical School, University of São Paulo, Ribeirão Preto, São Paulo, Brazil; b Division of Clinical Immunology, Department of Clinical Medicine, Ribeirão Preto Medical School, University of São Paulo, Ribeirão Preto, São Paulo, Brazil

## Abstract

Pemphigus are blistering autoimmune diseases related with genetic and environmental factors. Here we describe *HLA* genotyping in pemphigus patients. First, we review the *HLA* class I/II data on pemphigus reported in Brazilian samples and then present the *HLA* class I (-*A*, -*B*, -*C*) and class II (-*DRB1*, -*DQA1*, -*DQB1*) alleles related to susceptibility/resistance to pemphigus by comparing 86 patients with pemphigus foliaceus, 83 patients with pemphigus vulgaris, and 1592 controls from the northeastern region of the state of São Paulo, Southeastern Brazil. The data presented here are related to the manuscript “Differential *HLA* class I and class II associations in Pemphigus Foliaceus and Pemphigus Vulgaris patients from a prevalent Southeastern Brazilian region” Brochado et al. (2016) [1].

## Specifications Table

**Table:** 

Subject area	*Biology*
More specific subject area	*Dermatology, Immunology, and Genetics*
Type of data	*Tables*
How data was acquired	*The reviewing of the literature was made by using the Pubmed, and the HLA typing by using PCR-SSOP method*
Data format	*Analyzed*
Experimental factors	*DNA blood samples from pemphigus patients and controls*
Experimental features	*HLA class I and II typing was performed using commercial kits (One Lambda Inc., Canoga Park, CA)*
Data source location	*Northeastern region of the state of São Paulo, Southeastern Brazil*
Data accessibility	*Data is with this article*

## Value of the data

• The literature review regarding *HLA* class I/II data on pemphigus is shown in tables comparing different studied Brazilian populations.
• The northeastern region of the state of São Paulo, Southeastern Brazil, is prevalent for both clinical forms of pemphigus–pemphigus foliaceus and pemphigus vulgaris, enabling a comparative study.
• *HLA* class I/II frequencies are detailed comparing pemphigus foliaceus and pemphigus vulgaris patients from the same endemic region.

## 1 Data

Table 1, Table 2 describe the *HLA* class I and II data related to susceptibility/resistance to pemphigus foliaceus and pemphigus vulgaris in reviewed Brazilian reports. Table 3, Table 4, Table 5, Table 6, Table 7, Table 8 show the *HLA* class I (-*A*, -*B*, -*C*) and class II (-*DRB1*, -*DQA1*, -*DQB1*) profile performed in pemphigus foliaceus and pemphigus vulgaris patients from Southeastern Brazil.

## 2 Experimental design, materials, and methods

A summary of Brazilian data regarding associations between *HLA* and pemphigus was obtained in PubMed. A hundred and sixty-nine patients followed up at the University Hospital of the Ribeirão Preto Medical School of the University of São Paulo, Brazil, were evaluated. Eighty-six and 83 patients exhibited PF and PV, respectively. The control group consisted of 1592 healthy individuals living in the northeastern region of the state of São Paulo, Southeastern Brazil. *HLA* class I and II typing was performed at low/high resolution by using commercial kits, according to the manufacturer׳s protocol (One Lambda Inc., Canoga Park, CA). The allelic frequencies of the *HLA* class I and II genes were estimated by direct counting. Comparison of allele frequency among the groups was performed by using Fisher׳s exact test or the Chi-square test. Significant *P*-values were corrected by the number of alleles tested for each locus. The relative risk (RR) 95% was estimated. Statistical analysis was performed with SAS 9.3 (SAS Institute Inc, EUA) and Epi InfoTM 7.0 (CDC, USA) software. Values *P*≤0.05 were considered significant. All the participants provided an informed written consent to participate in this study. The local Ethics Committee (#12248/2010) approved this study.

## Figures and Tables

**Table 1:** Brazilian reports on pemphigus foliaceus associated alleles.

**References**		**Brazilian population**	**Patients/Controls**	DRB1*	P-value, RR or OR	DQA1*	*P*-value, RR	DQB1*	*P*-value, RR or OR
**Susceptibility – Pemphigus Foliaceus**									
PetzL-Erler et al. (1989) [2]	North to Southwest of the state of Paraná		48/74	01	Pc=3.3×10^−3^, RR=6.4				
				04	*P*=3.3×10^−3^, RR=3.3				
Moraes et al. (1991) [3]	State of São Paulo and Brasília city (Federal District)		38/50	01:02	Pc=0.002, RR=7.3				
Cerna et al. (1993) [4]	Xavante Indians – Central Brazil		10/74	04:04	Pc=0.03, RR=9.6				
Moraes et al. (1997) [5]	Terena Indians – state of Mato Grosso do Sul		20/66	04:04	Pc=0.022, OR=6.1			03:02	Pc=0.04, OR=5.2
Pavoni et al. (2003) [6]	State of Mato Grosso do Sul and Paraná		128/402	01	*P*<10^−6^, OR=7.4				
				01:01	*P*=0.042, OR=1.83				
				01:02	*P*<10^−6^, OR=10.36				
				01:03	*P*=0.025, OR=5.41				
				04	*P*<10^−6^, OR=2.66				
				04:04	*P*=4×10^−6^, OR=4.58				
				04:06	*P*=5.2×10^−6^, OR=35.85				
				04:10	*P*=0.046, OR=9.62				
				14:06	*P*=0.04, OR=4.04				
				16:01	*P*=0.017, OR=2.87				
Brochado et al. (2016) [1]	Northeastern region of the state of São Paulo		86/1592	01:01	Pc=0.0001, RR=2.18	01	Pc=0.02, RR=1.41	05:01	Pc=2.5×10^−10^, RR=2.95
				01:02	Pc=5.4e^−^^10^, RR=6.06	01:02	Pc=3.6×10^−3^, RR=2.3		
						03	Pc=0.01, RR=1.78		

**Protection – Pemphigus Foliaceus**									
Petzl-Erler et al. (1989) [2]	North/Southwest of the state of Paraná		48/74	07	Pc=9×10^−3^, RR=0.06			02:01	*P*=8.1×10^−3^, RR=0.27
Moraes et al. (1991) [3]	State of São Paulo and Brasília city (Federal District)		38/50					02:01	*P*=0.006, RR=0.04
								06:02	Pc=0.042, OR=0.15
Pavoni et al. (2003) [6]	States of Mato Grosso do Sul and Paraná		128/402	03:01	*P*=8.7×10^−4^, OR=0.23				
				07:01	*P*<10^−6^, OR=0.09				
				08	*P*=1×10^−3^, OR=0.27				
				08:01	*P*=7.2×10^−3^, OR=0.07				
				11	*P*<10^−6^, OR=0.09				
				11:01	*P*<10^−6^, OR=0.05				
				11:04	*P*=0.03, OR=0.15				
				14:02	*P*=0.018, OR=0.09				
				15	*P*=0.019, OR=0.51				
Brochado et al. (2016) [1]	Northeastern region of the state of São Paulo			11:01	Pc=0.027, RR=0.07	02:01	Pc=0.03, RR=0.34	03:01	Pc=0.0002; RR=0.39
				13:01	Pc=0.027, OR=0.20	05	Pc=1.08×10^−5^, RR=0.28	06:03	Pc=0.023, RR=0.2

RR=Relative risk, OR=odds ratio, Pc=*P*-values were corrected by the number of alleles tested for each locus.

**Table 2:** Brazilian reports on pemphigus vulgaris associated alleles.

**References**	**Brazilian population**	**Patients/Controls**	DRB1*	*P*-value, OR or RR	DQA1*	*P*-value, OR	DQB1*	*P*-value, RR
**Susceptibility – Pemphigus Vulgaris**								
Weber et al. (2011) [7]	Southeastern region of the state of São Paulo	36/162	04:02	OR=44.6				
			08:04	OR=18.6				
			14	OR=4.8				
Brochado et al. (2016) [1]	Northeastern region of the state of São Paulo	82/1592	04:02	Pc=5.4×10^−10^, RR=12.54	03	Pc=0.01, OR=2.04	03:02	Pc=2.5×10^−10^, RR= 2.95
			08:04	Pc=5.4×10^−5^, RR=6	03:01	Pc=3.6×10^−4^, OR=4	05:03	Pc=0.02, OR=2.74
			14:01	Pc=5.4×10^−10^, RR=7				
			14:04	Pc=5.4×10^−4^, RR=16.64				

**Protection – Pemphigus Vulgaris**								
Brochado et al. (2016) [1]	Northeastern region of the state of São Paulo	82/1592	07:01	Pc=0.027, RR=0.28			06:02	Pc=0.0075, RR=0.19

RR=Relative risk, OR=odds ratio, Pc=*P*-values were corrected by the number of alleles tested for each locus.

**Table 3:** Allelic *HLA-A* frequencies among Brazilian pemphigus foliaceus and pemphigus vulgaris patients as compared to controls.

HLA-A*	**Controls (*n*=1592)**	**Pemphigus foliaceus (*****n*****=83)**	**Pemphigus vulgaris (*****n*****=83)**
	***n* (%)**	***n* (%)**	***n* (%)**
**01**	291 (9.14)	12 (7.23)	21 (12.65)
**02**	1028 (32.29)	31 (18.67)^a^	38 (22.89)
**03**	277 (8.70)	18 (10.84)	9 (5.42)
**04**	1 (0.03)	0	0
**06**	2 (0.06)	0	0
**11**	126 (3.96)	16 (9.64)^b^	11 (6.63)
**23**	127 (3.99)	8 (4.82)	4 (2.41)
**24**	324 (10.18)	16 (9.64)	18 (10.84)
**25**	38 (1.19)	2 (1.20)	4 (2.41)
**26**	86 (2.70)	5 (3.01)	13 (7.83)^c^
**29**	114 (3.58)	4 (2.41)	2 (1.20)
**30**	163 (5.12)	14 (8.43)	11 (6.63)
**31**	149 (4.68)	6 (3.61)	4 (2.41)
**32**	95 (2.98)	3 (1.81)	5 (3.01)
**33**	73 (2.29)	11 (6.63)^d^	6 (3.61)
**34**	18 (0.57)	3 (1.81)	1 (0.60)
**36**	13 (0.41)	1 (0.60)	1 (0.60)
**66**	17 (0.53)	3 (1.81)	2 (1.20)
**68**	205 (6.44)	8 (4.82)	13 (7.83)
**69**	1 (0.03)	0	0
**74**	34 (1.07)	5 (3.01)	1 (0.60)
**80**	2 (0.06)	0	2 (1.20)

RR=Relative Risk, CI=confidence interval.

a *P*=2.10^−4^, RR=0.57, 95% CI=0.42–0.80.

b *P*=0.04, RR=2.43, 95% CI=1.5–4.0.

c *P*=0.02, RR=2.89, 95% CI=1.65–5.08.

d *P*=0.04, RR=2.89, 95% CI=1.56–5.34.

**Table 4:** Allelic HLA-B* frequencies among Brazilian pemphigus foliaceus and pemphigus vulgaris patients as compared to controls.

HLA-B*	**Controls (*****n*****=1592)**	**Pemphigus foliaceus (*****n*****=83)**	**Pemphigus vulgaris (*****n*****=82)**
	***n* (%)**	***n* (%)**	***n* (%)**
**07**	229 (7.19)	13 (7.83)	5 (3.05)
**08**	111 (3.49)	8 (4.82)	2 (1.22)
**13**	35 (1.10)	3 (1.81)	4 (2.44)
**14**	161 (5.06)	23 (13.86)^a^	4 (2.44)
**15**	379 (11.90)	12 (7.23)	5 (3.05)^b^
**18**	185 (5.81)	3 (1.81)	5 (3.05)
**27**	45 (1.41)	3 (1.81)	4 (2.44)
**35**	444 (13.94)	19 (11.45)	26 (15.85)
**37**	25 (0.79)	3 (1.81)	2 (1.22)
**38**	60 (1.88)	2 (1.20)	12 (7.32)^c^
**39**	113 (3.55)	11 (6.63)	9 (5.49)
**40**	124 (3.89)	10 (6.02)	8 (4.88)
**41**	31 (0.97)	1 (0.60)	1 (0.61)
**42**	33 (1.04)	2 (1.20)	3 (1.83)
**44**	333 (10.46)	14 (8.43)	23 (14.02)
**45**	45 (1.41)	4 (2.41)	3 (1.83)
**47**	5 (0.16)	0	0
**48**	21 (0.66)	3 (1.81)	0
**49**	68 (2.14)	1 (0.60)	4 (2.44)
**50**	75 (2.36)	4 (2.41)	4 (2.44)
**51**	330 (10.36)	8 (4.82)	13 (7.93)
**52**	49 (1.54)	3 (1.81)	2 (1.22)
**53**	55 (1.73)	3 (1.81)	8 (4.88)
**55**	33 (1.04)	2 (1.20)	4 (2.44)
**56**	4 (0.13)	1 (0.60)	0
**57**	92 (2.89)	6 (3.61)	10 (6.10)
**58**	86 (2.70)	4 (2.41)	1 (0.61)
**73**	4 (0.13)	0	1 (0.61)
**81**	8 (0.25)	0	1 (0.61)
**82**	1 (0.03)	0	0

RR=Relative Risk, CI=confidence interval.

a *P*=6×10^−4^, RR=2.74, 95% CI=1.82–4.12.

b *P*=0.003, RR=0.26, 95% CI=0.10–0.61.

c *P*=0.003, RR=3.88, 95% CI=2.13–7.07.

**Table 5:** Allelic *HLA-C* frequencies among Brazilian pemphigus foliaceus and pemphigus vulgaris patients as compared to controls.

HLA-C*	**Controls (*****n*****=1305)**	**Pemphigus foliaceus (*****n*****=83)**	**Pemphigus vulgaris (*****n*****=82)**
	***n* (%)**	***n* (%)**	***n* (%)**
**01**	55 (2.11)	3 (1.81)	5 (3.05)
**02**	176 (6.74)	15 (9.04)	10 (6.10)
**03**	278 (10.65)	14 (8.43)	11 (6.71)
**04**	475 (18.20)	27 (16.27)	32 (19.51)
**05**	144 (5.52)	8 (4.82)	13 (7.93)
**06**	212 (8.12)	14 (8.43)	14 (8.54)
**07**	578 (22.15)	33 (19.88)	24 (14.63)
**08**	135 (5.17)	17 (10.24)	5 (3.05)
**12**	155 (5.94)	10 (6.02)	21 (12.80)^a^
**14**	79 (3.03)	0	4 (2.44)
**15**	111 (4.25)	13 (7.83)	11 (6.71)
**16**	144 (5.52)	7 (4.22)	10 (6.10)
**17**	52 (1.99)	3 (1.81)	4 (2.44)
**18**	16 (0.61)	2 (1.20)	0

RR=Relative Risk, CI=confidence interval

a *P*=0.01, RR=2.16, 95% CI=1.40–3.30.

**Table 6:** Allelic *HLA-DRB1* frequencies among Brazilian pemphigus foliaceus and pemphigus vulgaris patients as compared to controls.

***HLA-DRB1⁎***	**Controls (*n*=1592)**	**Pemphigus foliaceus (*****n*****=86)**	**Pemphigus vulgaris (*****n*****=82)**
	***n* (%)**	***n* (%)**	***n* (%)**
**01:01**	150 (4.7)	23 (13.4)^a^	6 (3.7)
**01:02**	113 (3.6)	37 (21.5)^b^	3 (1.8)
**01:03**	22 (0.7)	1 (0.6)	0
**03:01**	247 (7.8)	10 (5.8)	4 (2.4)
**03:02**	25 (0.8)	3 (1.7)	2 (1.2)
**04**	0	2 (1.2)	0
**04:01**	79 (2.5)	3 (1.7)	2 (1.2)
**04:02**	65 (2.0)	4 (2.3)	42 (25.6)^c^
**04:03**	38 (1.2)	2 (1.2)	4 (2.4)
**04:04**	88 (2.8)	10 (5.8)	5 (3.1)
**04:05**	63 (2.0)	4 (2.3)	1 (0.6)
**04:06**	8 (0.3)	1 (0.6)	0
**04:07**	27 (0.9)	1 (0.6)	0
**04:08**	21 (0.7)	2 (1.2)	0
**04:10**	2 (0.1)	0	0
**04:11**	54 (1.7)	7 (4.1)	2 (1.2)
**07:01**	342 (10.7)	6 (3.5)	5 (3.1)^d^
**08:01**	71 (2.2)	0	2 (1.2)
**08:02**	34 (1.1)	3 (1.7)	1 (0.6)
**08:03**	10 (0.3)	0	0
**08:04**	42 (1.3)	2 (1.2)	13 (7.9)^e^
**08:07**	25 (0.8)	1 (0.6)	0
**09:01**	41 (1.3)	4 (2.3)	0
**10:01**	43 (1.4)	1 (0.6)	1 (0.6)
**11**	0	0	1 (0.6)
**11:01**	258 (8.1)	1 (0.6)^f^	9 (5.5)
**11:02**	72 (2.3)	1 (0.6)	5 (3.1)
**11:03**	31 (1.0)	0	0
**11:04**	138 (4.3)	1 (0.6)	4 (2.4)
**11:06**	1 (0.03)	0	0
**11:13**	1 (0.03)	0	0
**11:18**	1 (0.03)	0	0
**12:01**	35 (1.1)	3 (1.7)	1 (0.6)
**12:02**	3 (0.1)	0	0
**13:01**	274 (8.6)	3 (1.7)^g^	4 (2.4)
**13:02**	158 (5.0)	2 (1.2)	3 (1.8)
**13:03**	55 (1.7)	0	2 (1.2)
**13:05**	2 (0.1)	0	0
**13:06**	1 (0.03)	0	0
**13:21**	1 (0.03)	0	0
**13:23**	1 (0.03)	0	0
**13:31**	1 (0.03)	0	0
**14:01**	70 (2.2)	0	25 (15.2)^h^
**14:02**	35 (1.1)	1 (0.6)	3 (1.8)
**14:04**	7 (0.2)	2 (1.2)	6 (3.7)^i^
**14:06**	7 (0.2)	1 (0.6)	0
**14:09**	1 (0.03)	0	0
**15:01**	194 (6.1)	12 (7.0)	2 (1.2)
**15:02**	24 (0.8)	0	2 (1.2)
**15:03**	79 (2.5)	5 (2.9)	0
**15:04**	1 (0.03)	0	0
**15:11**	1 (0.03)	0	0
**16:01**	66 (2.1)	10 (5.8)	2 (1.2)
**16:02**	56 (1.8)	3 (1.7)	2 (1.2)

RR=relative risk, CI=confidence interval.

a *P*=1×10^−4^, RR=2.83, 95% CI=1.88–4.28.

b *P*=5×10^−10^, RR=6.06, 95% CI=4.32–8.49.

c *P*=5.4×10^−10^, RR=12.54, 95% CI=8.79–17.88.

d *P*=0.027, RR=0.28, 95% CI=0.12–0.67.

e *P*=5.4×10^−5^, RR=6.0, 95% CI=3.29–10.97.

f *P*=0.027, RR=0.07, 95% CI=0.01–0.50.

g *P*=0.027, RR=0.20, 95% CI=0.06–0.62.

h *P*=5.4×10^−10^, RR=7.21, 95% CI=4.72–10.99.

i *P*=5.4×10^−4^, RR=16.64, 95% CI=5.65–48.95.

**Table 7:** Allelic *HLA-DQA1* frequencies among Brazilian pemphigus foliaceus and pemphigus vulgaris patients as compared to controls.

***HLA-DQA1⁎***	**Controls (*****n*****=1312)**	**Pemphigus foliaceus (*****n*****=86)**	**Pemphigus vulgaris (*****n*****=82)**
	***n* (%)**	***n* (%)**	***n* (%)**
**01**	760 (29.0)	70 (40.7)^a^	47 (28.7)
**01:02**	155 (5.9)	24 (14.0)^b^	8 (4.9)
**01:03**	130 (5.0)	3 (1.7)	4 (2.4)
**01:06**	3 (0.11)	0	0
**01:07**	4 (0.15)	2 (1.2)	0
**01:09**	1 (0.04)	0	0
**02:01**	267 (10.2)	6 (3.5)^c^	6 (3.7)
**03**	318 (12.1)	37 (21.5)^d^	36 (22.0)^e^
**03:01**	88 (3.4)	5 (2.9)	20 (12.2)^f^
**04**	1 (0.04)	0	0
**04:01**	146 (5.6)	7 (4.1)	10 (6.1)
**04:03**	2 (0.1)	0	0
**04:04**	2 (0.1)	0	0
**05**	499 (19.0)	9 (5.2)^g^	27 (16.5)
**05:01**	201 (7.7)	9 (5.2)	5 (3.1)
**05:02**	2 (0.1)	0	0
**05:10**	33 (1.3)	0	1 (0.6)
**06:01**	12 (0.5)	0	0

RR=relative risk, CI=confidence interval.

a *P*=0.02, RR=1.41, 95% CI=1.16–1.69.

b *P*=3.6×10^−3^, RR=2.36, 95% CI=1.58–3.52.

c *P*=0.03, RR=0.34, 95% CI=0.15–0.75.

d *P*=0.01, RR=1.78, 95% CI=1.31–2.40.

e *P*=0.01, RR=1.81, 95% CI=1.33–2.46.

f *P*=4×10^−4^, RR=3.64, 95% CI=2.29–5.75.

g *P*=1.08×10^−5^, RR=0.28, 95% CI=0.14–0.52.

**Table 8:** Allelic *HLA-DQB1* frequencies among Brazilian pemphigus foliaceus and pemphigus vulgaris patients as compared to controls.

HLA-DQB1*	**Controls (*n*=1411)**	**Pemphigus foliaceus (*n*=86)**	**Pemphigus vulgaris (*n*=82)**
	***n* (%)**	***n* (%)**	***n* (%)**
**02:01**	233 (8.26)	9 (5.23)	4 (2.44)
**02:02**	260 (9.21)	6 (3.49)	5 (3.05)
**03**	2 (0.07)	0	1 (0.61)
**03:01**	626 (22.18)	15 (8.72)^a^	30 (18.29)
**03:02**	297 (10.52)	27 (15.70)	51 (31.10)^b^
**03:03**	75 (2.66)	4 (2.33)	1 (0.61)
**03:04**	3 (0.11)	0	0
**03:05**	1 (0.04)	0	1 (0.61)
**03:19**	13 (0.46)	0	2 (1.22)
**04**	0	1 (0.58)	1 (0.61)
**04:01**	3 (0.11)	0	0
**04:02**	170 (6.02)	12 (6.98)	9 (5.49)
**05**	0	7 (4.07)	22 (13.41)
**05:01**	328 (11.62)	59 (34.30)^c^	10 (6.10)
**05:02**	91 (3.22)	11 (6.40)	3 (1.83)
**05:03**	69 (2.45)	1 (0.58)	11 (6.71)^d^
**05:05**	1 (0.04)	0	0
**06**	8 (0.28)	0	0
**06:01**	19 (0.67)	0	3 (1.83)
**06:02**	263 (9.32)	16 (9.30)	3 (1.83)^e^
**06:03**	226 (8.01)	3 (1.74)^f^	4 (2.44)
**06:04**	99 (3.51)	1 (0.58)	3 (1.83)
**06:09**	29 (1.03)	0	0
**06:11**	5 (0.18)	0	0
**16:02**	1 (0.04)	0	0

RR=relative risk, CI=confidence interval.

a *P*=2×10^−4^, RR=0.39, 95% CI=0.24–0.64.

b *P*=2.5×10^−10^, RR=2.95, 95% CI=2.3–3.80.

c *P*=2.5×10^−10^, RR=2.95, 95% CI=2.34–3.71.

d *P*=0.02, RR=2.74, 95% CI=1.49–5.08.

e *P*=7.5×10^−3^, RR=0.19, 95% CI=0.06–0.60.

f *P*=0.02, RR=0.22, 95% CI=0.07–0.67.
