# Supplementary material for: Data on HLA class I/II profile in Brazilian pemphigus patients
Source: Data Brief. 2016 Jun 3;8:364–74. doi: 10.1016/j.dib.2016.05.077 (PMC4909822; doi:10.1016/j.dib.2016.05.077)
Supplement: Supplementary file 1 — Supplementary material [file mmc1.docx]

***HLA* class I/II profile in Brazilian pemphigus patients**

Maria José Franco Brochado^a^, Daniela Francisca Nascimento^a^, Neifi Hassan Saloum Deghaide^b^, Eduardo Antonio Donadi^b^, Ana Maria Roselino^a*^

1. Division of Dermatology, Department of Clinical Medicine, Ribeirão Preto Medical School, University of São Paulo, Ribeirão Preto, São Paulo state, Brazil
2. Division of Clinical Immunology, Department of Clinical Medicine, Ribeirão Preto Medical School, University of São Paulo, Ribeirão Preto, São Paulo state, Brazil

***Correspondence to:**

University Hospital, Ribeirão Preto Medical School, University of São Paulo, São Paulo, Brazil. Avenida Bandeirantes, 3900 - Ribeirão Preto, SP, Brazil, CEP: 14049-900. Phone number: +55 (16) 36022447; Fax number: +55 (16) 36021522; E-mail: [amfrosel@fmrp.usp.br](mailto:amfrosel@fmrp.usp.br)

**Conflict of Interest**: "The authors state no conflict of interest."

**Submission declaration and verification:** This manuscript has not been published previously, it is not under consideration for publication elsewhere, and this publication was approved by all authors.
